# Data on the anti-tumor effects of *Selaginella tamariscina* extract and amentoflavone combined with doxorubicin in mice

**DOI:** 10.1016/j.dib.2017.05.023

**Published:** 2017-05-17

**Authors:** Chang Gun Lee, Eun Ha Lee, Cheol-Ho Pan, Kyungsu Kang, Ki-Jong Rhee

**Affiliations:** a Department of Biomedical Laboratory Science, College of Health Sciences, Yonsei University, Wonju 26493, Republic of Korea; b Systems Biotechnology Research Center, Korea Institute of Science and Technology, Gangneung 25451, Republic of Korea; c Department of Biological Chemistry, University of Science and Technology (UST), Daejeon 34113, Republic of Korea

**Keywords:** Amentoflavone, Anti-tumor, Doxorubicin, *Selaginella tamariscina*

## Abstract

Here, we report animal experimental data associated with the article entitled “AKR1B10-inhibitory *Selaginella tamariscina* extract and amentoflavone decrease the growth of A549 human lung cancer cells in vitro and in vivo“ (Jung et al., 2017) [1]. We tested the synergistic anti-tumor effects of *Selaginella tamariscina* extract and amentoflavone combined with doxorubicin hydrochloride in a nude mouse xenograft model of A549 human lung cancer cells. In our experiment, *Selaginella tamariscina* extract and amentoflavone were administered orally; and doxorubicin hydrochloride was injected intraperitoneally. We expect our preliminary data will be helpful to the development of the anticancer agent using *Selaginella tamariscina* extract or amentoflavone.

**Specifications Table**

**Table:** 

Subject area	Biology
More specific subject area	Natural products science
Type of data	Figure
How data was acquired	Animal experiment
Data format	Analyzed
Experimental factors	A549 human lung cancer cells were cultured *in vitro*, and then, subcutaneously injected to the right lateral flank of athymic female nude mice (BALB/c).
Experimental features	Ethanolic extract of *Selaginella tamariscina* and amentoflavone were administered orally; and doxorubicin hydrochloride was administered intraperitoneally.
Data source location	Wonju and Gangneung, South Korea
Data accessibility	Data are available in this article

**Value of the data**

• Effects of *Selaginella tamariscina* extract and amentoflavone on the tumor growth were evaluated in the nude mouse xenograft model of A549 cells.
• Synergistic anti-tumor effects *of Selaginella tamariscina* extract and amentoflavone combined with doxorubicin hydrochloride were evaluated *in vivo*.
• This data may provide some information for the development of anticancer agents using phytochemicals.

## 1 Data

**Fig. 1:**
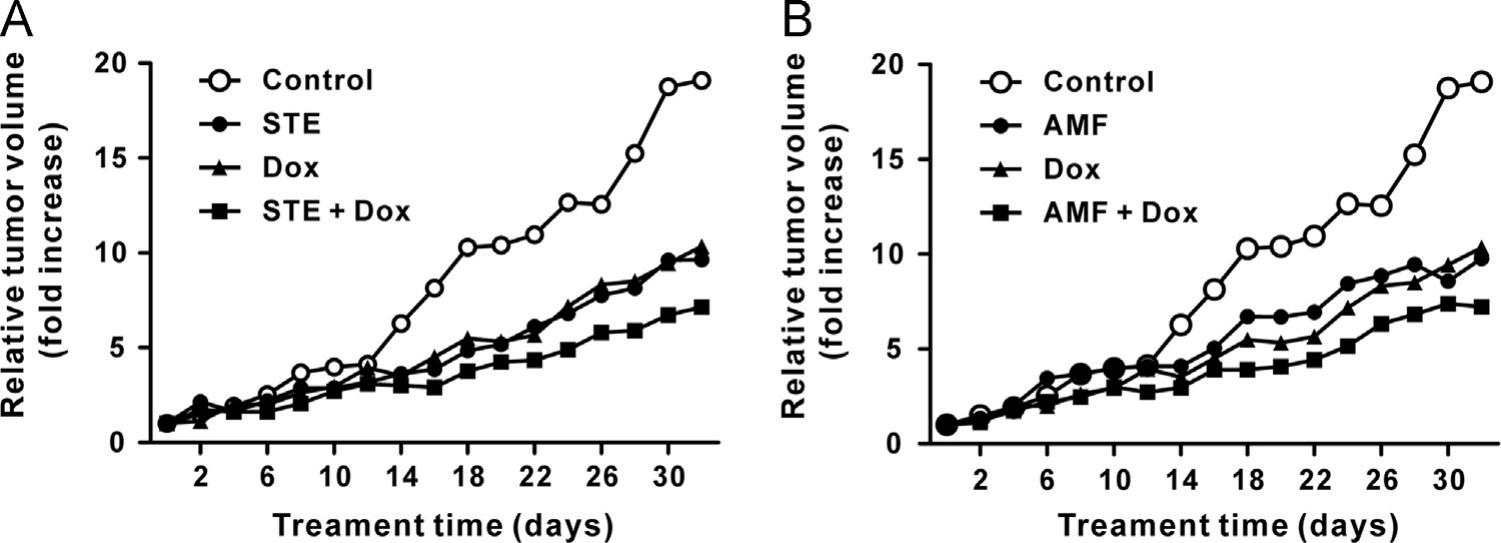
Combinatorial effects of the *Selaginella tamariscina* extract (STE, A) and amentoflavone (AMF, B) on the anti-tumor effects of doxorubicin in a nude mouse xenograft model of A549 cells. The median tumor volume was monitored for 32 days. STE (1000 mg/kg) and amentoflavone (50 mg/kg, AMF) were administered via oral gavage every two days. Doxorubicin hydrochloride (Dox, 1 mg/kg) was administered intraperitoneally every three days. The co-treatment group was treated with STE (1000 mg/kg) plus doxorubicin hydrochloride (1 mg/kg) (A) or AMF (50 mg/kg) plus doxorubicin hydrochloride (1 mg/kg) (B). The relative tumor volume (RTV) was calculated as follows: RTV=V_t_/V_0_. The curves of relative tumor volume from the vehicle control mice and the doxorubicin-treated mice were plotted in both figures (A, B).

Here, we described the synergistic anti-tumor effects of *Selaginella tamariscina* extract and amentoflavone combined with the doxorubicin hydrochloride treatment in mice. The combinatorial effects of *Selaginella tamariscina* extract (Fig. 1A) or amentoflavone (Fig. 1B) on the anti-tumor action of doxorubicin hydrochloride were evaluated. There is no statistical difference between the single treatment of doxorubicin and the combinatorial treatment of these phytochemicals and doxorubicin.

## 2 Experimental design, materials and methods

### 2.1 Reagents

Dimethyl sulfoxide (DMSO) and doxorubicin hydrochloride were purchased from Sigma-Aldrich (St. Louis, MO, USA). Amentoflavone was purchased from Tokyo Chemical Industry (Tokyo, Japan). Ethanolic extract of *S. tamariscina* (P. Beauv.) Spring was prepared as described in the associated article [1].

### 2.2 Nude mouse xenograft experiments

To estimate the combinatorial effect of *S. tamariscina* extract or amentoflavone on the anti-tumor effects of doxorubicin *in vivo*, we performed nude mouse xenograft experiments with a small number of mice (*n*=5 for each group). Seven-week-old athymic female nude mice (BALB/c) were purchased from Raonbio (Yongin, Korea). The mice were maintained in a filter-top cage with a 12-h light/12-h dark cycle. Sterile food pellets (Teklad-certified irradiated global 18% protein rodent diet 2018S; Harlan Teklad, Madison, WI, USA) and autoclaved water were provided *ad libitum*. A549 human lung adenocarcinoma cells were cultured in Dulbecco׳s Modified Eagle׳s Medium (DMEM) supplemented with heat-inactivated 10% (v/v) fetal bovine serum and antibiotics. Tumor xenografts were established by subcutaneous injection of A549 cells (1×10^7^ cells) in 200 μL of serum-free media into the right lateral flank. The tumor volumes were measured using calipers and the following formula: *V*=0.5×length×width^2^. When the tumor volume reached approximately 200 mm^3^, mice were randomized into treatment groups consisting of 5 animals per group. Mice were administered *S. tamariscina* extract (1000 mg/kg, 1% DMSO in phosphate-buffered saline; PBS) or amentoflavone (50 mg/kg, 1% DMSO in PBS) via oral gavage every two days. Doxorubicin hydrochloride (1 mg/kg in PBS) was intraperitoneally administered every three days. Control group mice were given either 1% DMSO in PBS oral gavage or PBS intraperitoneal injection alone. The tumor volume was measured every two days for 32 days. The relative tumor volume (RTV) was calculated using the following formula: RTV=*V*_t_ (tumor volume on the measured day)/*V*_0_ (the initial tumor volume on day 0) as described previously [2].

### 2.3 Statistical analysis

Statistical analyses were performed by one-way analysis of variance followed by the Tukey׳s multiple comparison test using GraphPad Prism 6 software (GraphPad Software, La Jolla, CA, USA). *p*<0.05 was considered statistically significant.
