# Supplementary material for: Data on the anti-tumor effects of Selaginella tamariscina extract and amentoflavone combined with doxorubicin in mice
Source: Data Brief. 2017 May 17;13:162–5. doi: 10.1016/j.dib.2017.05.023 (PMC5451183; doi:10.1016/j.dib.2017.05.023)
Supplement: Supplementary file 1 — Supplementary material [file mmc1.pdf]

AUTHOR DECLARATION

We declare that there is no conflict of interest.

We confirm that the manuscript has been read and approved by all named authors and that there are no other persons who satisfied the criteria for authorship but are not listed. We further confirm that the order of authors listed in the manuscript has been approved by all of us.

We understand that the Corresponding Author is the sole contact for the Editorial process (including Editorial Manager and direct communications with the office). He/she is responsible for communicating with the other authors about progress, submissions of revisions and final approval of proofs. We confirm that we have provided a current, correct email address which is accessible by the Corresponding Author and which has been configured to accept email from (kskang@kist.re.kr)

Signed by the Corresponding Author on behalf of the all other authors

[Kyungsu Kang, 29-March-2017]
